# Radiological Diagnosis and Advances in Imaging of Vertebral Compression Fractures

**DOI:** 10.3390/jimaging10100244

**Published:** 2024-09-28

**Authors:** Kathleen H. Miao, Julia H. Miao, Puneet Belani, Etan Dayan, Timothy A. Carlon, Turgut Bora Cengiz, Mark Finkelstein

**Affiliations:** 1 Department of Diagnostic, Molecular, and Interventional Radiology, Icahn School of Medicine at Mount Sinai, New York, NY 10029, USA; 2 Department of Radiology, University of Chicago Medicine, Chicago, IL 60637, USA; 3 Department of Radiology, University of Miami Miller School of Medicine, Miami, FL 33136, USA; 4 Department of Radiology, Wake Forest University School of Medicine, Winston-Salem, NC 27157, USA; 5 Department of Radiology, NYU Grossman School of Medicine, New York, NY 10016, USA

**Keywords:** radiology, imaging, vertebral compression fracture, spine, diagnosis, treatment, prognosis

## Abstract

Vertebral compression fractures (VCFs) affect 1.4 million patients every year, especially among the globally aging population, leading to increased morbidity and mortality. Often characterized with symptoms of sudden onset back pain, decreased vertebral height, progressive kyphosis, and limited mobility, VCFs can significantly impact a patient’s quality of life and are a significant public health concern. Imaging modalities in radiology, including radiographs, computed tomography (CT), magnetic resonance imaging (MRI), and positron emission tomography (PET) studies and bone scans, play crucial and evolving roles in the diagnosis, assessment, and management of VCFs. An understanding of anatomy, and the extent to which each imaging modality serves to elucidate that anatomy, is crucial in understanding and providing guidance on fracture severity, classification, associated soft tissue injuries, underlying pathologies, and bone mineral density, ultimately guiding treatment decisions, monitoring treatment response, and predicting prognosis and long-term outcomes. This article thus explores the important role of radiology in illuminating the underlying anatomy and pathophysiology, classification, diagnosis, treatment, and management of patients with VCFs. Continued research and advancements in imaging technologies will further enhance our understanding of VCFs and pave the way for personalized and effective management strategies.

## 1. Introduction

Globally, vertebral compression fractures (VCFs) are a common clinical condition characterized by the collapse or deformity of vertebral bodies, predominantly affecting the thoracic and lumbar spine. VCFs can lead to debilitating pain, decreased mobility, and increased mortality rates, making accurate diagnosis and effective management essential to improving the lives of patients. In the United States, VCFs pose a significant health burden, occurring in approximately 1.4 million Americans annually [1].

Osteoporosis, a major risk factor for VCFs, affects over 10 million adults in the United States [2]. It was estimated that an untreated 14.0 million in the European Union plus Switzerland and the UK were eligible for the treatment of osteoporosis [3]. In the Canadian Longitudinal Study on Aging, 7.8% of the study cohort, including 30,097 participants aged 45 to 85 years, had osteoporosis [4]. As the aging population continues to grow worldwide, it is projected that the prevalence of VCFs will continue to rise. This in turn contributes to an ever-growing estimated annual cost of VCFs, with the estimated cost of VCFs secondary to osteoporosis expected to rise.

Females have a higher VCF incidence compared to males, primarily due to the higher prevalence of osteoporosis after menopause. In younger patients, the most common causes of VCFs are trauma-related, like motor vehicle accidents, falls, and sports injuries. Furthermore, a research study analyzed a large cohort that showed those with radiographic evidence of VCFs have an increased rate of mortality, especially from pathological causes like cancer and pulmonary disease like COPD and pneumonia [5].

Sedentary behavior, lack of weight-bearing exercises, smoking, excessive alcohol consumption, and poor nutrition negatively affect bone health, increasing the risk of VCFs. Additionally, decreased musculature is associated with non-traumatic VCFs in the elderly [6]. Family history, genetics, long-term use of certain medications like corticosteroids, and medical conditions like multiple myeloma and cancers are risk factors. Osteoporosis-related VCFs are insufficiency fractures from normal stress on abnormal bone that can occur during low-impact activities as opposed to stress fractures, which are abnormal stress on normal bone. Furthermore, VCFs can lead to altered spinal alignment and biomechanics, which increase the risk of subsequent fractures [7].

VCFs are associated with chronic pain, reduced mobility, functional decline, and increased risk of subsequent fractures [8]. The pain is often described as sharp, stabbing, or aching and may worsen with movement, weight-bearing, or certain activities. Furthermore, the loss of vertebral height can result in respiratory compromise, spinal deformities, and decreased pulmonary function [8]. Severe VCFs that cause spinal cord compression can lead to neurological symptoms like weakness in the limbs, difficulty walking, or bowel and bladder dysfunction.

Imaging plays a pivotal role in the identification, diagnosis, characterization, and management of VCFs. Differential diagnoses for VCFs involves distinguishing these fractures from conditions with similar presenting symptoms, including back pain and deformities such as scoliosis. Osteoporosis, trauma, pathologic fractures from metastases or multiple myeloma, infections like osteomyelitis, and degenerative changes can present with similar symptoms. This article highlights techniques such as radiographs (XR), computed tomography (CT) including dual-energy computed tomography (DECT), magnetic resonance imaging (MRI), and positron emission tomography (PET) studies and bone scans, which are essential in diagnosing VCFs, evaluating and assessing the severity, underlying causes, and complications.

The novelty of this article on the radiological diagnosis and advances in imaging of VCFs lies in its comprehensive analysis of the latest imaging modalities and their contributions in diagnosis, treatment, management, and patient outcomes. By comparing and evaluating the strengths, limitations, and clinical utility of each imaging modality, this review highlights key contributions of radiologic imaging in the management of VCFs in the clinical setting.

The literature search for this article was performed through a comprehensive and systematic approach to ensure that all relevant, high-quality articles were included from multiple research databases, including PubMed and Google Scholar. A wide range of peer-reviewed research articles, meta-analyses, clinical trials, and systematic review articles published in the last 25 years in the fields of radiology, neurosurgery, and orthopedic surgery were incorporated with keyword search terms related to radiologic imaging and diagnosis of VCFs. Recent and relevant publications were thoroughly evaluated in this rigorous search and references within the articles were additionally reviewed to ensure comprehensive inclusion. By examining the different imaging modalities commonly employed in the evaluation of VCFs, this study will shed light on the evolving role of radiology in optimizing patient care.

## 2. Anatomy

VCFs are common spinal injuries that involve the collapse or compression of vertebral bodies typically in the thoracic or lumbar regions. The vertebral body bears the majority of the load, providing support and stability to the spine. It is estimated that 60–75% of VCFs occur in the thoracolumbar junction at T12-L2 and approximately 30% at L2-L5 [9]. The fractures may be single or multilevel.

### 2.1. Three-Column Spinal Concept of Spinal Fractures

**Figure 1:**
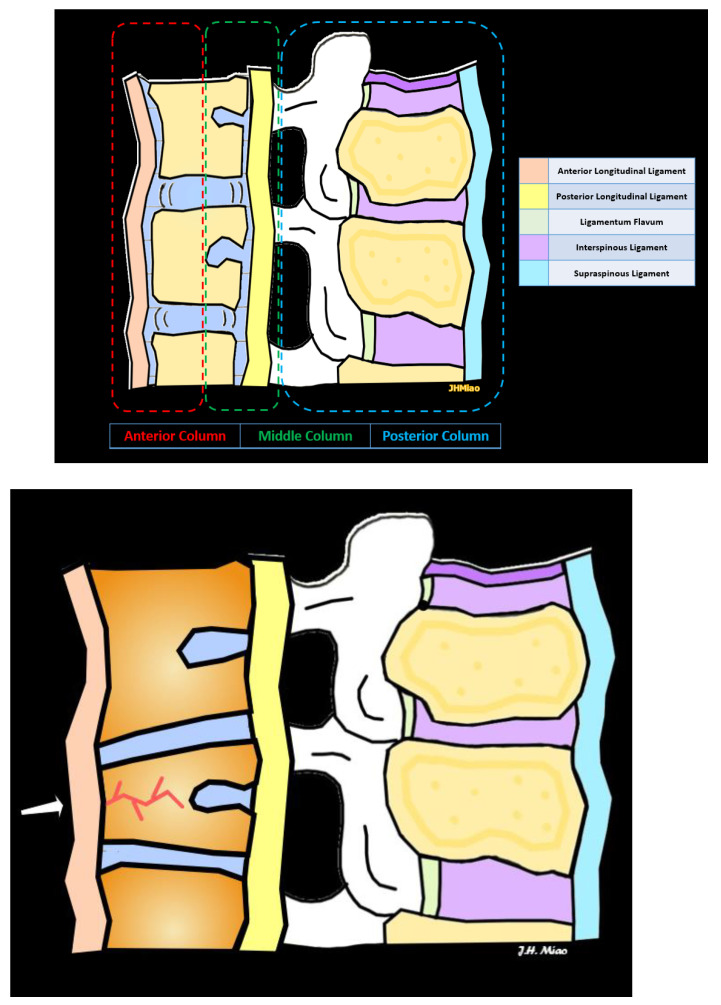
Illustration of the spinal anatomy (**top**), including anterior column, middle column, posterior column, anterior longitudinal ligament, vertebral column, posterior longitudinal ligament, interspinous ligament, and supraspinous ligament. In this illustration of a vertebral compression fracture (**bottom**), the arrow shows afracture line, anterior wedging, and mild loss of vertebral body height.

The three-column spinal concept is a fundamental framework used in the evaluation of spinal fractures and often used to assess stability. The three columns, including the anterior column, middle column, and posterior column, encompass different structural components, each contributing to the overall stability of the vertebral column [10] (Figure 1).

The anterior column consists of the anterior longitudinal ligament (ALL), anterior annulus fibrosus, and anterior half of the vertebral body (Figure 1). VCF fractures involving the anterior column often result from axial loading mechanisms and can be associated with compromised anterior stability. The ALL is a strong fibrous band that runs along the anterior aspect of the vertebral bodies from the base of the skull to the sacrum, connecting adjacent vertebral bodies. The ALL provides stability and limits excessive extension of the spine. In VCFs, the ALL may remain intact or get stretched or torn, depending on fracture severity.

The middle column encompasses the posterior half of the vertebral body, posterior annulus fibrosus, and posterior longitudinal ligament (PLL). It provides a crucial stabilizing effect in both the sagittal and coronal planes. Fractures involving the middle column, including burst fractures and flexion-distraction injuries, often result from excessive axial loading or a combination of flexion and distraction forces. These fractures may exhibit loss of vertebral height, retropulsion of bony fragments into the spinal canal, and disruption of the posterior ligamentous complex.

The posterior column comprises the posterior elements of the vertebrae, including the laminae, facet joints, spinous processes, pedicles, and ligamentum flavum. It provides essential stability against shear and rotational forces. Fractures involving the posterior column are often associated with significant instability, resulting from hyperextension or hyperflexion injuries.

### 2.2. Types of Vertebral Compression Fractures

**Figure 2:**
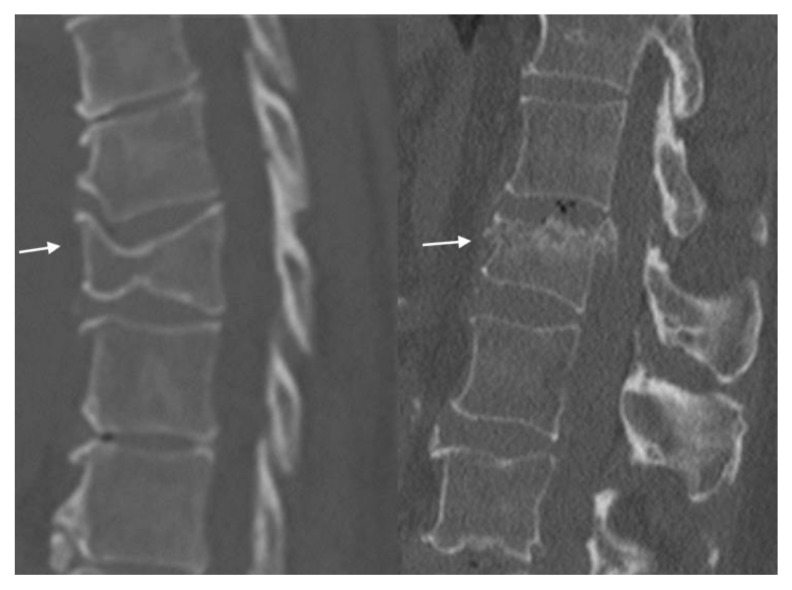
In this sagittal CT (**left**), the arrow shows a T8 vertebral body wedge compression fracture with moderate loss of vertebral height. In this sagittal CT (**right**), the arrow shows an acute to subacute burst compression fracture of the superior endplate of L1 with approximately 40% height loss. The bones are diffusely demineralized.

Wedge fractures involve a collapse of the anterior vertebral body, typically caused by low-energy axial loading, such as a fall from standing height or osteoporosis, and lead to wedge-shaped deformity. The posterior elements of the vertebrae, including the pedicles and laminae, are usually unaffected while the anterior longitudinal ligament may or may not be affected. These VCFs can often be managed conservatively, especially when asymptomatic. Wedge compression fractures can range from mild, moderate, and severe loss of vertebral body height (Figure 2).

Burst fractures are characterized by the vertebral body collapsing in multiple fragments and is a more severe disruption of the vertebral body, involving both the anterior and posterior portions (Figure 2). This leads to a loss of vertebral body height and a more fragmented appearance. The vertebral body shatters and disperses into several fragments that may encroach upon and potentially compromise the spinal canal, often associated with significant neurological deficits.

## 3. Classification of Fractures

### 3.1. The Genant Classification System

**Figure 3:**
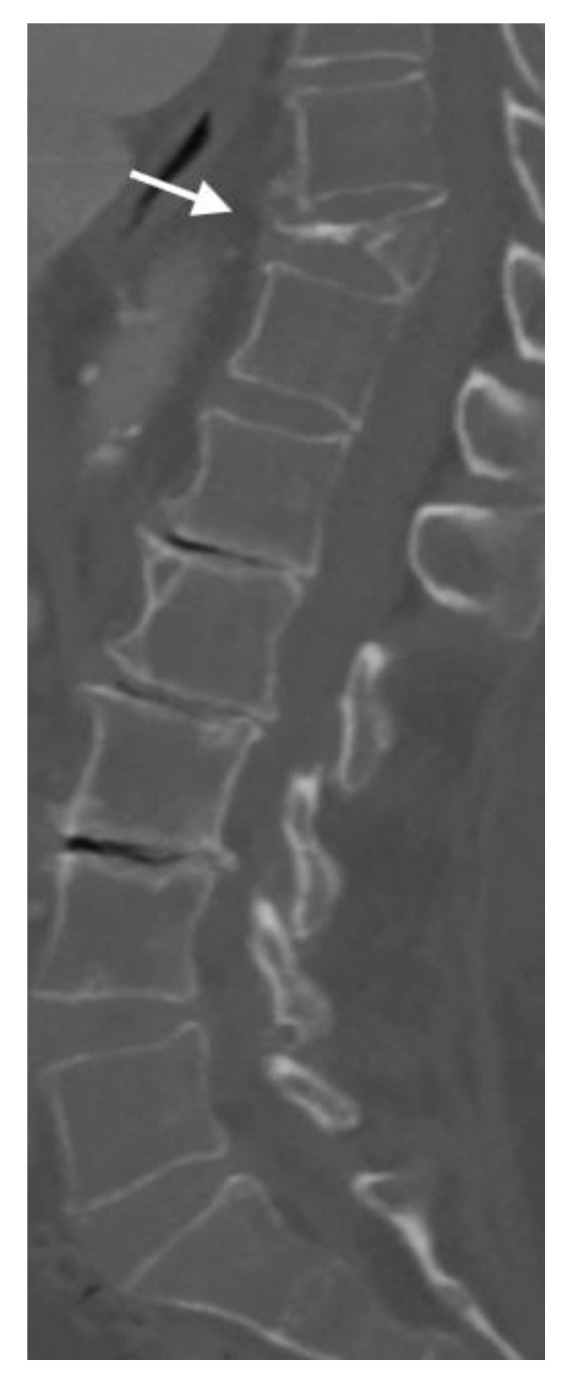
In this sagittal CT, the arrow shows severe T11 vertebral compression fracture, Grade 3 according to Genant classification. The bones are diffusely demineralized.

The Genant classification system assesses the severity of VCFs based on the vertebral body height loss and the presence of endplate deformity [11] (Table 1). In Figure 3, a Grade 3 compression deformity is demonstrated with severe loss of vertebral body height in T12.

### 3.2. The Denis Classification System

The Denis classification system categorizes thoracolumbar fractures based on the involvement of the three columns of the spine, anterior, middle, and posterior, providing a general overview of the fracture pattern and stability of the fracture [10] (Table 2).

### 3.3. Association for the Study of Internal Fixation/Arbeitsgemeinschaft für Osteosynthesefragen (AO) Spine Classification System

The Association for the Study of Internal Fixation/Arbeitsgemeinschaft für Osteosynthesefragen (AO) Spine Classification system considers the fracture morphology and the integrity of the posterior ligamentous complex. This system provides detailed information about the fracture pattern and merging imaging, clinical, and prognostic factors to guide treatment decisions [12] (Table 3).

### 3.4. Thoracolumbar Injury Classification and Severity Score (TLICS) System

The Thoracolumbar Injury Classification and Severity Score (TLICS) system incorporates injury morphology, neurological status, and the presence of additional injuries, to guide treatment decisions [7,9] (Table 4).

The AO spine and TLICS classification systems are the most frequently used in clinics because of their structured, evidence-based, and clinically relevant frameworks for evaluating VCFs, especially for guiding treatment decisions. Both systems combine radiologic imaging with clinical findings such as neurological deficits and spinal stability, providing a comprehensive evaluation of the fracture. Finally, both TLICS and AO spine systems are universally accepted and supported by extensive research, making them reliable classification systems in the clinical setting.

## 4. Imaging Modalities and Features

### 4.1. Radiographs

Radiographs are often the initial imaging study performed when VCFs are suspected or incidentally found. Radiographs are useful for assessing overall alignment, detecting fractures, and evaluating for signs of vertebral collapse or deformity [13]. They are also used for monitoring healing progress over time. Key radiological findings play a crucial role in the diagnosis and evaluation of VCFs, including loss of vertebral height, wedge or burst vertebral fractures, cortical fracture lines, decreased intervertebral disc space, sclerosis and callus formation, and associated spinal alignment changes. Plain radiographs have difficulty distinguishing potential etiologies of VCFs, such as osteoporosis, metastatic disease, or high-energy trauma, as well as clarifying the acuity of VCFs.

**Figure 4:**
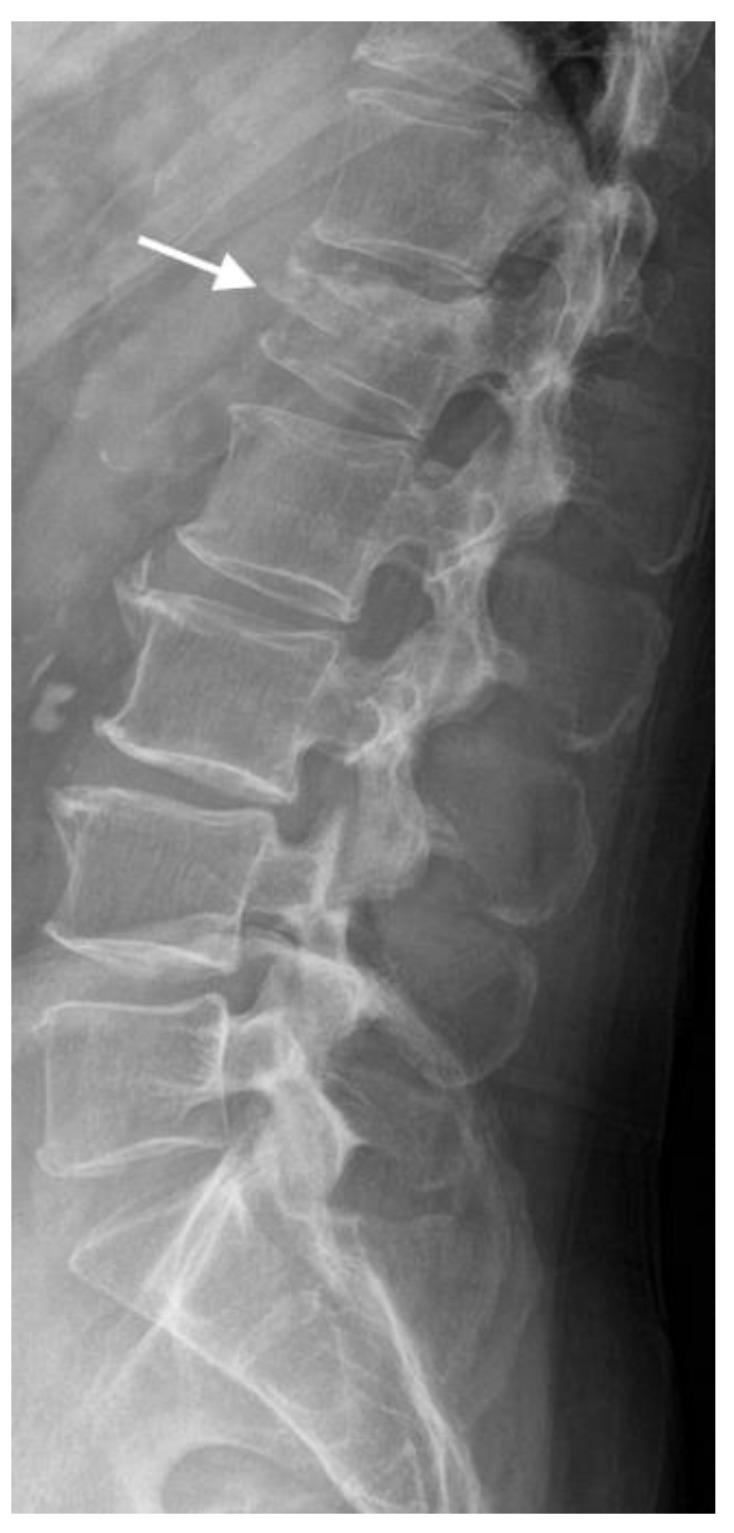
In this lateral radiograph of the lumbar spine, the arrow demonstrates compression fracture of the superior endplate of L1 with mild to moderate vertebral height loss.

VCFs often cause a wedge-shaped deformity of the vertebral body due to the collapse of the anterior vertebral body, resulting in a characteristic trapezoidal appearance on radiographs. In severe cases, the vertebral body may exhibit a burst appearance with loss of normal vertebral contours. Radiographs may show evidence of fracture lines within the vertebral body, which appear as radiolucent lines crossing the vertebral body or as cortical disruption (Figure 4).

VCFs may lead to a reduction in the intervertebral disc space between the affected vertebra and its adjacent vertebrae. This can be observed as a decreased distance between the endplates of the vertebrae on radiographs. Over time, VCFs may undergo a healing process, resulting in the formation of new bone and sclerosis at the fracture site. This manifests as increased density or thickening of the trabecular bone in the affected vertebral body. Furthermore, VCFs can cause changes in spinal alignment, such as an increase in thoracic kyphosis or the development of a compensatory lumbar lordosis.

### 4.2. CT Scans

CT imaging provides detailed three-dimensional evaluation of the extent, characteristics, and associated findings of VCF fractures, especially for complex fractures [11]. Typical radiological findings observed on CT imaging in VCFs include fracture line and vertebral body collapse, loss of cortical integrity, retropulsion and spinal canal stenosis, and posterior element involvement.

In acute fractures, CT imaging usually demonstrates the fracture line within the vertebral body, which appears as a disruption in the normal bony architecture. The fracture line may be linear or more complex, such as a wedge or burst fracture. CT can accurately assess the degree of vertebral body collapse or height loss, providing quantitative measurements of anterior, middle, and posterior vertebral body heights. Furthermore, CT imaging allows for the assessment of cortical disruption of VCFs. The presence of marginal spurs or paravertebral osteophytes can be observed adjacent to the fractured vertebral body.

**Figure 5:**
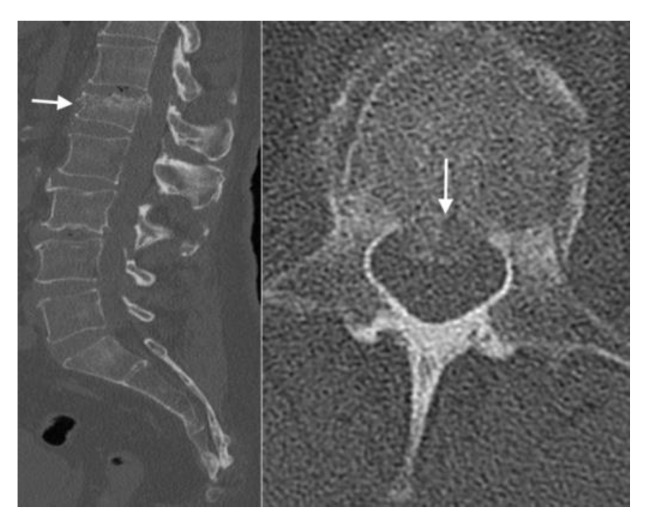
In this sagittal CT (**left**) of the spine, the arrow shows acute to subacute burst compression fracture of the superior endplate of L1 with approximately 40% height loss. The bones are diffusely demineralized. In this axial CT (**right**) of the L1 vertebral body, the arrow shows retropulsion of fragments with moderate spinal canal narrowing and flattening of the thecal sac.

Additionally, CT imaging can evaluate retropulsion of bone fragments into the spinal canal (Figure 5). The presence of retropulsed fragments can result in spinal canal compromise and potential compression of the spinal cord or nerve roots. CT enables precise measurements of the spinal canal diameter to assess the degree of stenosis and potential neurological implications.

VCFs can involve the posterior elements of the vertebrae, including the pedicles, laminae, and spinous processes. CT imaging can identify fractures or disruptions of these posterior elements, which may contribute to spinal instability or neurologic compromise. Evaluation of the posterior elements is crucial in assessing the need for surgical intervention. CT imaging can reveal soft tissue injuries, such as paraspinal hematoma or edema, which may indicate an acute fracture.

Importantly, CT imaging allows for 3D reconstruction, enhancing visualization and understanding of the fracture pattern, fracture fragments, and associated spinal anatomy. This aids in surgical planning, particularly for procedures such as vertebral augmentation or spinal fusion.

**Figure 6:**
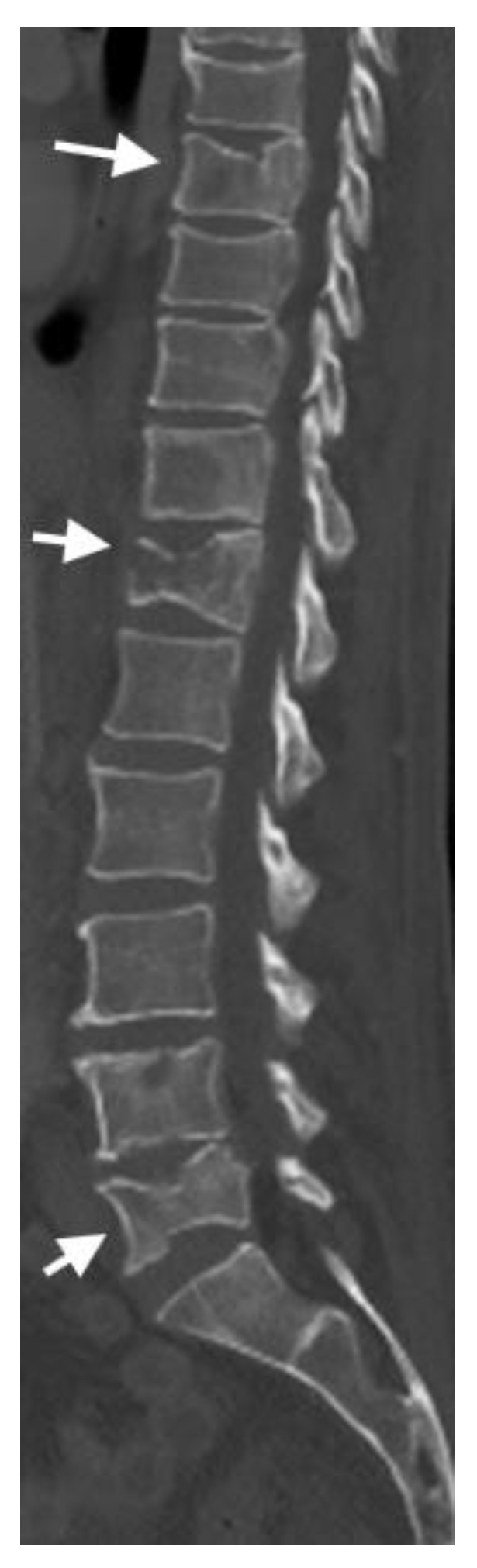
In this sagittal CT, the arrows show multifocal lytic lesions throughout the thoracolumbar spine. Multilevel, mild, chronic compression deformities of several vertebrae in this patient with metastatic melanoma.

CT imaging enables the identification of subtle fractures that may be missed on conventional radiography, particularly in osteoporotic fractures with minimal collapse. CTs can detect pathologic VCFs resulting from an underlying pathological condition, such as metastatic cancer, multiple myeloma, primary bone tumors, infection, or metabolic bone diseases [9] (Figure 6).

Additionally, dual-energy computed tomography (DECT) plays an important role in distinguishing between acute and chronic VCFs by evaluating bone marrow edema. Acute fractures are often associated with bone marrow edema, suggesting recent injury. DECT can detect increased water content within the bone marrow by using two different X-ray energy levels, which enhance contrast between water, bone, and fat. DECT creates virtual non-calcium images that subtract the dense calcified bone and therefore highlights soft tissue changes, such as edema. Compared with radiographs and conventional CTs, the benefits of DECT include detection of bone marrow edema and acuity of fractures. DECT also serves as a faster alternative to MRI for evaluating edema when MRI is contraindicated or unavailable. However, limitations of DECT include exposing patients to radiation and less sensitivity for evaluating soft tissue changes.

### 4.3. MRI Scans

MRI aids in the accurate characterization of VCFs, assessment of spinal stability, and evaluation of potential complications, guiding treatment decisions and monitoring healing progress. Typical radiological findings observed on MRI in VCFs include fracture line and vertebral body edema, bone marrow edema, loss of vertebral body height, disc changes and endplate involvement, spinal cord and nerve root compression, and associated soft tissue injuries. The collapsed vertebral body appears flattened or wedged on sagittal images, indicating the severity of the compression, which can be measured on MRI.

**Figure 7:**
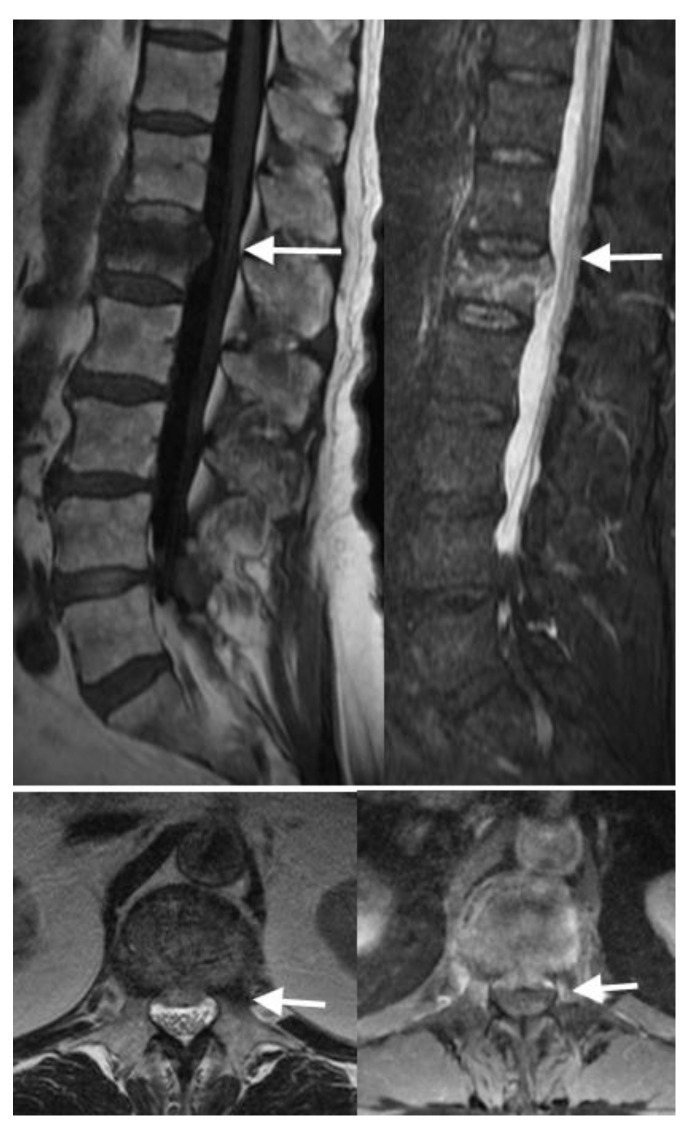
In these sagittal MRIs, the arrows show acute to subacute burst compression fracture of the superior endplate of L1 with accompanying T1 hypointense (**top left**)/STIR hyperintense (**top right**) marrow signal abnormality. In the axial T2 at this level (**bottom left**), the arrow shows 0.6 cm retropulsion of the superior endplate contributing to moderate spinal canal stenosis at this level, with crowding of the cauda equina nerve roots. In the axial post-contrast T1 (**bottom right**), the arrow shows heterogeneous enhancement which is likely reactive and expected.

**Figure 8:**
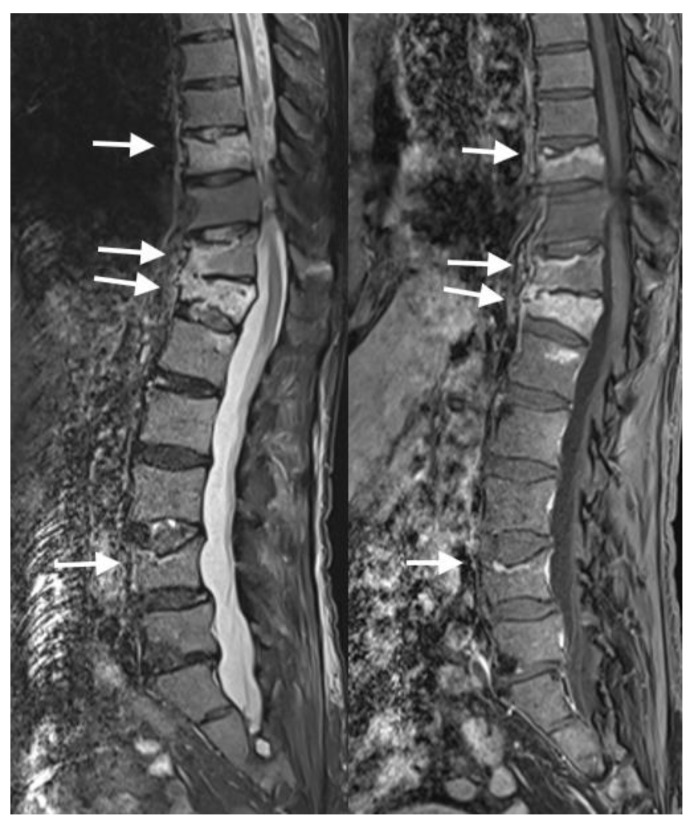
In these sagittal MRIs with STIR (**left**) and T1 fat saturated post contrast (**right**), the arrows show benign compression fractures of T9, T11, T12, and L4 with varying degrees of edema and enhancement. The enhancement is an expected finding and does not necessarily imply a pathologic fracture in the absence of other imaging signs.

MRI can visualize the fracture line within the vertebral body as an area of signal abnormality. In T1-weighted images, VCFs appear hypointense, indicating a loss of normal trabecular bone structure. On T2-weighted images or short tau inversion recovery (STIR) sequences, VCFs typically show hyperintense signals, reflecting edema or hemorrhage within the vertebral body (Figure 7). Bone marrow edema is indicative of the acute phase of the fracture and reflects increased vascularity and inflammatory processes within the bone marrow. MRI’s high sensitivity to edema allows for the detection of subtle changes in bone marrow signal intensity. This aids in differentiating acute fractures from chronic ones and monitoring the healing process [14] (Figure 8).

**Figure 9:**
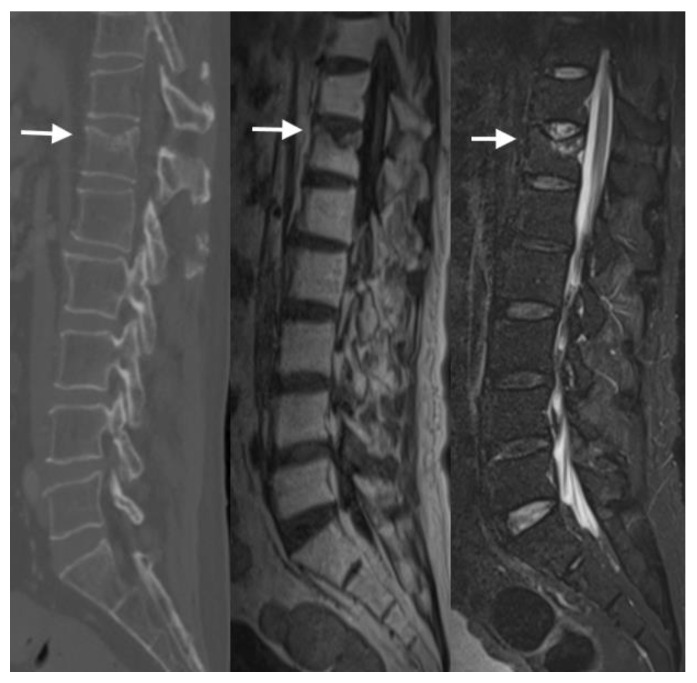
In the sagittal CT (**left**), T1 MRI (**middle**), and STIR MRI (**right**), the arrows show mild chronic superior T12 endplate compression deformity and loss of height with accompanying T1 hypointense/STIR hyperintense enhancing marrow signal abnormality. Slight STIR hyperintensity is seen along the superior T12 vertebral body reflecting edema, which is significantly improved from the prior scan.

Unlike acute fractures, chronic VCFs generally show resolution of edema on MRI, where the hyperintense signal on T2-weighted sequences decreases or resolves over time (Figure 9).

**Figure 10:**
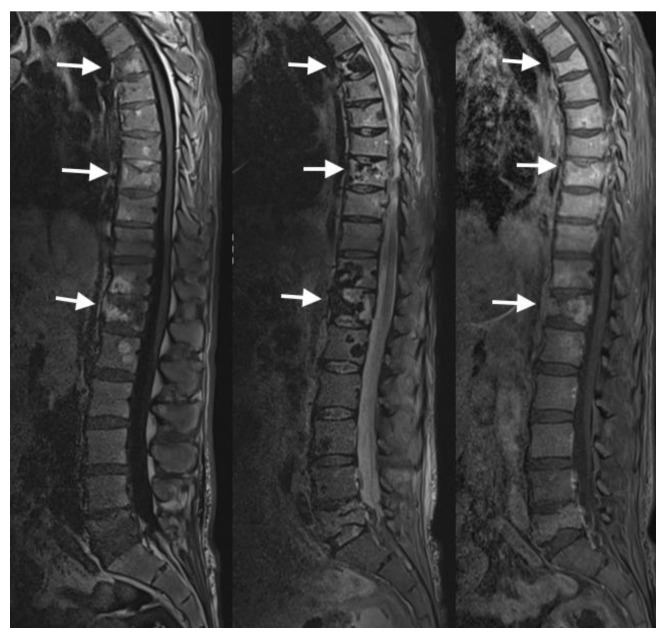
In the sagittal MRI sequences of T1, STIR, and post-contrast T1 from left to right, the arrows show multiple marrow replacing lesions throughout the osseous structures consistent with extensive metastatic disease in this melanoma patient. Multilevel chronic pathological compression fractures within the spine without significant retropulsion or spinal cord compression.

MRI can be especially helpful for distinguishing between benign and pathologic vertebral fractures [15]. MRI features of pathologic VCFs may include marrow replacement within the affected vertebral body and may involve multiple levels of the spine (Figure 10). Pathologic VCFs can exhibit soft tissue abnormalities, such as paraspinal soft tissue masses or tumor infiltration. Additionally, posterior convex morphology of the vertebral body is very characteristic for malignant pathologic VCFs [16].

**Figure 11:**
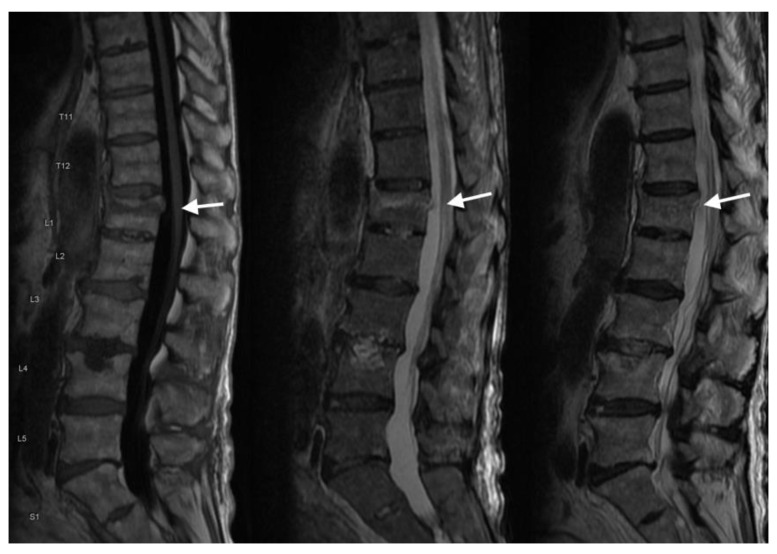
In the sagittal MRIs, the arrows show T1 (**left**), STIR (**middle**), and T2 (**right**) sequences demonstrating a recent L1 compression fracture with approximately 25% height loss. Very mild retropulsion of fracture fragments with trace spinal canal stenosis. No spinal cord/conus signal abnormality at this level.

Additionally, MRI enables the evaluation of spinal cord and nerve root compression in VCFs, including narrowing or encroachment on the neural structures. Compression of the spinal cord or nerve roots may present with signal changes, such as hyperintensity on T2-weighted images, indicating edema or myelopathy depending on the chronicity and whether there is volume loss in the cord (Figure 11).

### 4.4. Nuclear Medicine

Nuclear medicine imaging, such as bone scans coupled with SPECT/CT and PET/CT, can provide unique information regarding the physiology and functionality of the bone metabolism, bone turnover, and inflammatory processes.

PET scans utilize unique radiotracers, such as 18F-fluorodeoxyglucose (FDG), accumulating in areas of increased metabolic activity via utilizing glucose metabolism. In VCFs, secondary to ongoing inflammatory processes, active fractures may exhibit increased FDG uptake, indicating a higher metabolic demand in the affected vertebral body. Other PET radiotracers that may be useful in assessment of VCFs is 18F-NaF, a bone-only agent predominantly used for bone metastases.

Bone scans utilizing Technetium-99m (Tc) methyl-diphosphonate (Tc-MDP) are often used for detecting osseous metastatic disease in various cancer types (prostate, breast, lung, etc.). They can help identify VCFs and degenerative changes of the spine. Concomitant use of single photon emission computed tomography (SPECT) and CT along with the bone scan may increase sensitivity and specificity.

**Figure 12:**
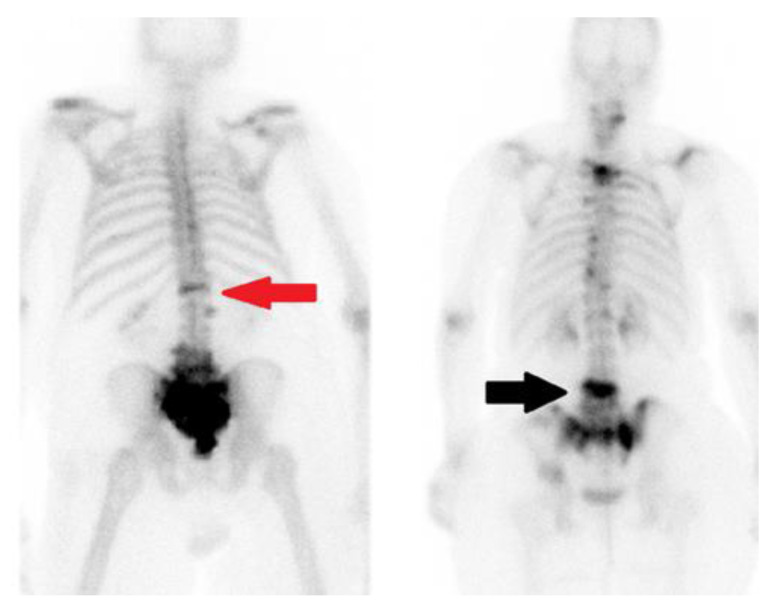
Two posterior view whole body Tc-MDP bone scans. The red arrow (**left**) shows a linear and well-defined uptake in the T12 vertebral body corresponding to a compression fracture in a prostate cancer patient. Black arrow (**right**) shows diffuse uptake in the entire vertebral body involved with metastatic breast cancer.

In VCFs, bone scans can reveal linear areas of uptake (Figure 12). The linear radiotracer uptake commonly seen in VCFs differs from the focal and more localized uptake seen in metastatic disease in bone scans.

**Figure 13:**
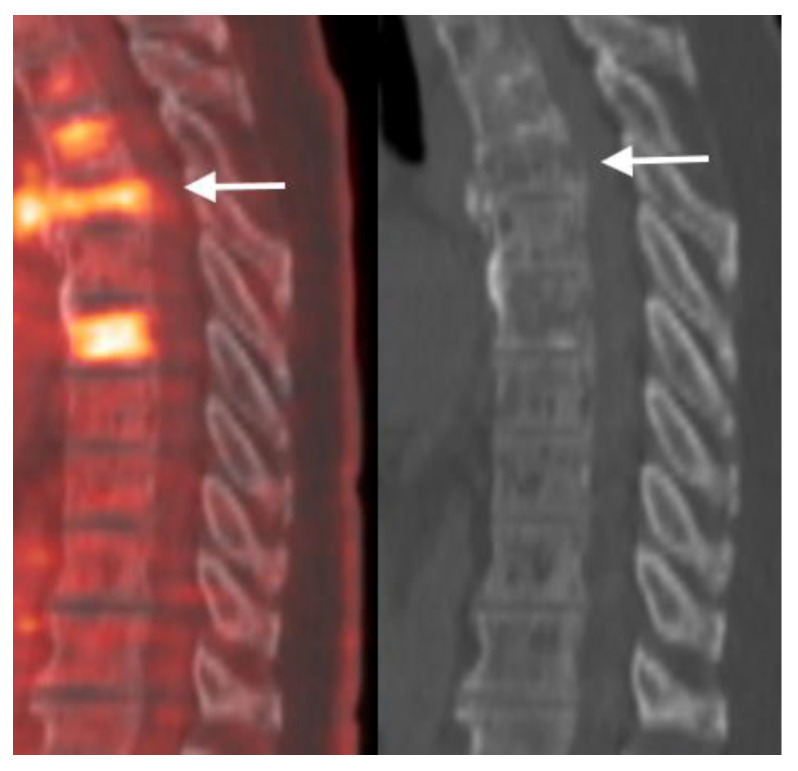
Sagittal view FDG fused PET-CT (**left**) and CT (**right**) of a patient with multiple myeloma. White arrow points to the T6 vertebral body compression fracture demonstrating linear FDG uptake. The T8 vertebral body is likely involved with disease and prone for compression fracture in the future. Note scattered lytic lesions throughout the spine.

PET scans can show increased uptake in metastatic spinal lesions and potential pathologic vertebral fractures. The appearance of diffuse radiotracer uptake in the whole vertebral body or linear radiotracer uptake along the upper or lower endplate may be an indicator for an impending VCF (Figure 13).

## 5. Prognosis

VCFs are common injuries that can significantly impact a patient’s quality of life. Prognostic evaluation plays a crucial role in determining the optimal management strategy and predicting long-term outcomes. Imaging techniques have proven to be valuable tools in assessing the prognosis of VCFs. The prognosis of VCFs depends on fracture severity, underlying medical conditions, patient age, and treatment provided. The VCF may progress over time although many VCFs heal spontaneously, and symptoms improve over time with conservative treatment measures. However, prognosis differs between VCFs caused by osteoporotic insufficiency fractures and traumatic fractures compared with pathologic VCFs, which have a poorer prognosis without treatment due to progression of the underlying disease.

Some VCFs lead to chronic pain, functional limitations, and decreased quality of life. A research review found that general health, physical disability, and pain relief are significantly improved with interventional procedures such as vertebroplasty and kyphoplasty for acute osteoporotic VCFs compared with medical management within the first 3 months after the interventional procedures [17]. Initial severe fractures with substantial height loss and spinal deformity are associated with severely increased and lasting pain, functional impairment, and poorer prognosis compared to mild fractures [18].

On MRI, persistent or progressive edema may suggest ongoing instability or delayed healing, leading to a less favorable prognosis. CT or MRI can detect spinal cord compression due to retropulsion of fractured vertebral fragments, which require immediate surgical intervention to prevent neurological complications.

Sequential imaging studies can assess fracture healing, restoration of vertebral body height, reduction in edema or inflammation, and stabilization of the spinal column. Follow-up imaging can guide decisions regarding the duration of immobilization, intervention, and transition to rehabilitation, ultimately influencing overall prognosis.

## 6. Treatment and Management

The treatment and management of VCFs depend on fracture severity, symptoms, underlying cause, and the overall health of the patient. Conservative management includes pain medication, rest and activity modification, back bracing, physical therapy, and calcium and vitamin D supplements [19]. Over-the-counter pain relievers such as nonsteroidal anti-inflammatory drugs may be used to manage pain. Opioid medications may be required, particularly in the acute phase and especially for cancer patients with pathologic fractures. Physical therapy can enhance mobility and functional abilities.

**Figure 14:**
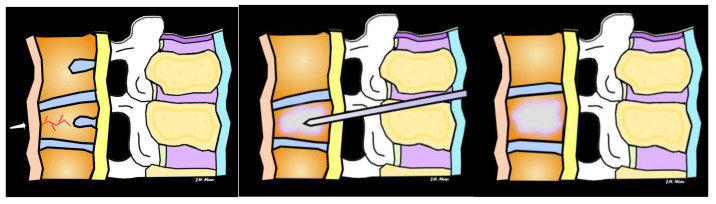
In the illustration of vertebroplasty, the arrow shows a vertebral compression fracture (**left**), the injection of bone cement into the fractured vertebra (**middle**), and the restoration of vertebral body height (**right**).

**Figure 15:**
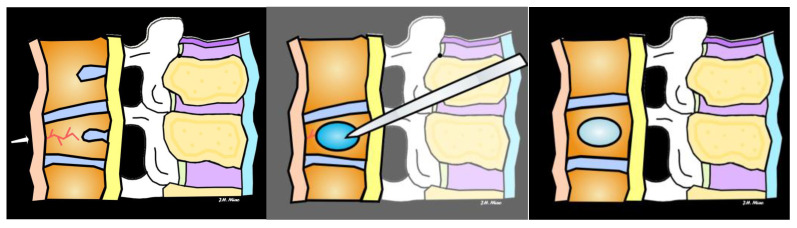
In the illustration of a kyphoplasty, the arrow shows a vertebral compression fracture (**left**), the insertion of a balloon into the fractured vertebra to create a cavity (**middle**), which is then filled with bone cement with restoration of vertebral body height (**right**).

**Figure 16:**
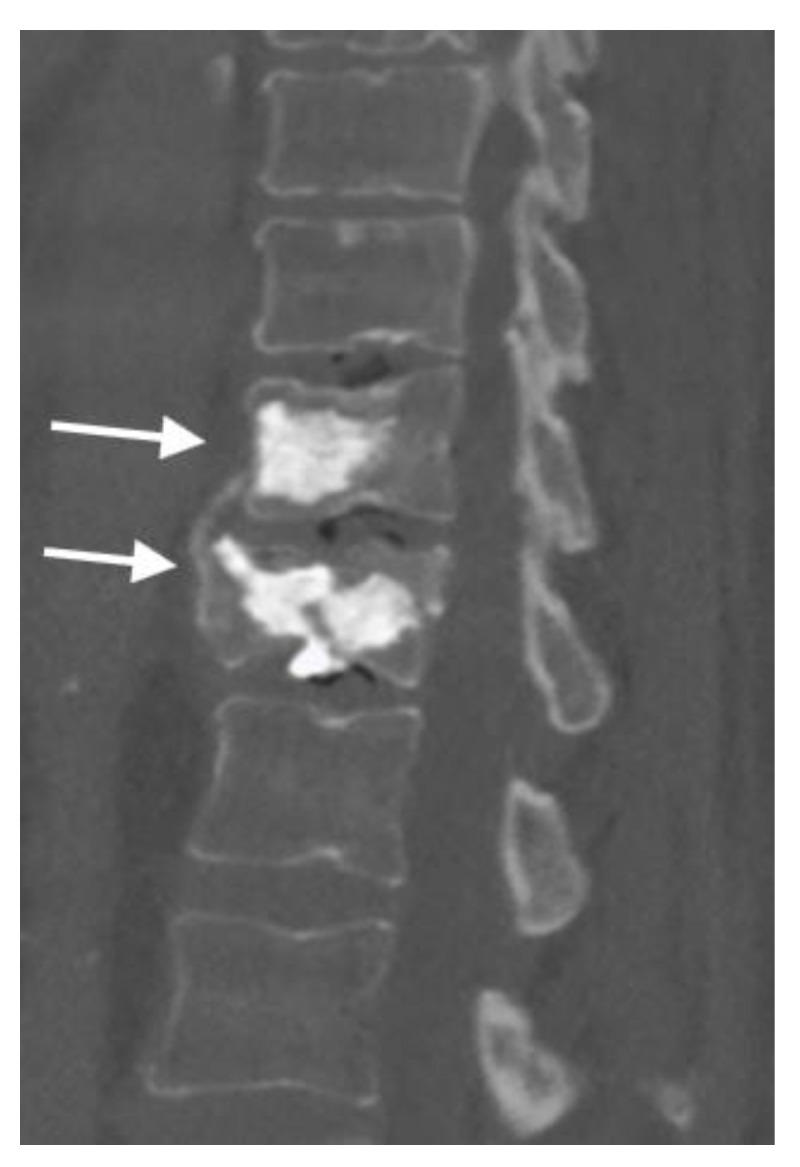
In the sagittal CT, the arrows show kyphoplasty for a T11 wedge compression fracture and acute traumatic comminuted T12 compression fracture, with mild height loss and no retropulsion.

Minimally invasive interventional procedures for VCFs include vertebroplasty and kyphoplasty to reduce pain and decrease or eliminate the need for pain medications like opioids. Vertebroplasty involves the injection of bone cement into the fractured vertebra to stabilize it and provide pain relief (Figure 14). It is typically performed under conscious sedation or monitored anesthesia care. In contrast to vertebroplasty, kyphoplasty first involves the insertion of a balloon into the fractured vertebra to create a cavity, which is then filled with bone cement (Figure 15 and Figure 16). This procedure aims to restore vertebral height and reduce deformity. A research review found that general health, physical disability, and pain relief outcomes are improved after vertebroplasty and kyphoplasty for osteoporotic VCFs compared with medical management within the first 3 months after the interventional procedures [20].

For VCFs associated with malignancy, external beam radiation therapy and thermal ablation can also be used to reduce local tumor burden and the associated pain. Surgery is considered in cases where conservative treatments and minimally invasive interventional procedures have failed, such as spinal fusion, vertebral augmentation with implants, or other stabilizing procedures.

The various treatment and management options including conservative management, vertebroplasty, kyphoplasty, and surgery are summarized in Table 5.

## 7. Conclusions

VCFs are common injuries that can have a significant impact on patients’ quality of life. Imaging techniques have greatly advanced the diagnosis and evaluation of VCFs, allowing for improved patient management and treatment outcomes.

Various imaging modalities, including radiographs, CT, MRI, PET, and bone scans, play a vital role in the diagnosis and assessment of VCFs, summarized in Table 6. Radiographs remain the initial screening tool, providing information on vertebral body height loss and deformity. CT offers detailed visualization of fracture morphology and DECT evaluates for bone marrow edema. MRI provides valuable insights into the presence of edema, inflammation, and associated soft tissue injuries. Nuclear medicine scans, such as PET or bone scans, aid in identifying underlying pathologies and assessing the extent of disease involvement. Furthermore, advancements in interventional procedures, such as vertebroplasty and kyphoplasty, and surgical interventions have enhanced patient quality of life and patient outcomes.

Advancements in imaging technology have facilitated the quantitative assessment of VCFs, leading to more objective and accurate evaluations. Advanced imaging techniques, such as high-resolution imaging and diffusion-weighted imaging (DWI), provide quantitative measurements of bone microarchitecture, water diffusion, and metabolic changes, respectively. These quantitative imaging biomarkers offer valuable information for fracture risk assessment, treatment response monitoring, and predicting future fracture occurrences [21]. Furthermore, DEXA scans assess bone mineral density and are crucial in providing vertebral fracture assessment and monitoring osteoporosis-related VCFs. Low BMD is a significant risk factor for VCFs, and DEXA scans aid in identifying patients at risk, guiding osteoporosis management, and assessing treatment response.

The integration of artificial intelligence (AI) and machine learning (ML) algorithms into VCF imaging research holds great promise. AI/ML techniques can aid in automated fracture detection, classification, and risk stratification. These algorithms can analyze large datasets and extract meaningful patterns from imaging and clinical data, providing decision support tools for accurate and efficient diagnosis. Furthermore, AI-based algorithms have the potential to assist in treatment planning and predicting treatment outcomes based on imaging features and patient-specific factors.

Imaging plays a crucial role in prognostic evaluation and treatment response assessment in VCFs. Treatment strategies for VCFs aim to relieve pain, restore function, prevent further fractures, and improve quality of life. Sequential imaging studies enable monitoring of fracture healing, restoration of vertebral body height, and stabilization of the spinal column, helping clinicians assess treatment response and guide further management decisions.

Imaging modalities in radiology thus play a critical role in assessing fracture severity and associated injuries, guiding treatment decisions, and monitoring treatment response, which enhance patient outcomes.

## 8. Future Directions

VCFs are a significant health concern, particularly among the globally aging population. Advances in imaging techniques have revolutionized the diagnosis and evaluation of VCFs, leading to improved patient care and outcomes. There are several potential future directions of imaging VCFS for further advancements in the field.

AI and ML have the potential to revolutionize VCF imaging. These technologies can aid in automated detection, characterization, and classification of VCFs on various imaging modalities [22]. For example, AI was used in a research study to develop a neural network for detecting VCFs on CTs [23]. AI algorithms can be used to analyze large datasets and assist in identifying subtle fractures, assessing fracture severity, and predicting treatment outcomes. ML algorithms can also integrate clinical, imaging, and genetic data to provide personalized risk assessment for VCFs and guide treatment decisions [24].

Quantitative imaging biomarkers hold promise for improving the objective assessment of VCFs. By utilizing advanced imaging techniques such as MRI or CT, quantitative measurements of bone density, trabecular microarchitecture, or bone composition can be obtained [25]. These biomarkers can provide valuable information on fracture risk assessment, monitoring treatment response, and predicting future fracture occurrences.

Emerging imaging techniques, such as high-resolution imaging or diffusion-weighted imaging (DWI), have the potential to provide additional insights into VCFs. High-resolution imaging can visualize subtle fractures and microstructural changes within the vertebral bodies [26]. DWI can assess water diffusion patterns and aid in differentiating between acute and chronic fractures and between benign and pathologic fractures.

The future of VCF imaging also includes advancements in image-guided interventions. Minimally invasive interventional procedures, such as vertebral augmentation (vertebroplasty or kyphoplasty), benefit from real-time imaging guidance using techniques like cone-beam CT or intraoperative MRI [27]. These imaging modalities can enhance accuracy, optimize needle placement, and provide immediate feedback during the procedure, improving patient outcomes.

AI, quantitative imaging biomarkers, advanced imaging techniques, image-guided interventions, and integration of imaging and clinical data are all promising directions for the future of VCFs and imaging. As research and technology continue to progress, the field of VCF imaging is poised to make significant strides in the prevention, diagnosis, and management of VCFs, improving the lives of patients and enhancing patient outcomes.

## Figures and Tables

**Table 1:** Genant classification system.

Grade	Description
Grade 0	Normal vertebral body.
Grade 1	Mild height reduction (<25%) or mild endplate deformity.
Grade 2	Moderate height reduction (25–40%) or moderate endplate deformity.
Grade 3	Severe height reduction (>40%) or severe endplate deformity.

**Table 2:** Denis classification system.

Type	Description
Type A	Anterior column only.
Type B	Middle column (including the posterior cortex of the vertebral body).
Type C	Posterior column (including the neural arch).

**Table 3:** AO spine classification system.

Type	Description
Type A	Simple fractures without much fragmentation.
Type A1	Minimal displacement, indicating minimal instability.
Type A2	More displacement than type A1 fractures, indicating a moderate level of instability.
Type A3	Extensive displacement and are the most unstable among type A fractures.
Type B	Partial articular fractures that involve the joint surface but do not extend through it completely.
Type B1	Involve a single articular surface and have a low level of instability.
Type B2	Affect two articular surfaces and exhibit moderate instability.
Type B3	Involve multiple articular surfaces and are highly unstable.
Type C	Complete articular fractures that extend through the joint surface. Highly unstable and often require surgical intervention.
Type C1	Extra-articular, and occur away from the joint itself.
Type C2	Involve a metaphyseal region and extend into the joint.
Type C3	Extend into the joint and affect both the metaphysis and diaphysis.

**Table 4:** TLICS classification system.

Severity Score	Description
Score 1–3	Nonoperative treatment recommended.
Score 4–7	Operative or nonoperative treatment based on neurological status and injury characteristics.
Score ≥8	Operative treatment recommended.

**Table 5:** Treatment and management options for VCFs.

	Description	Indications	Benefits
Conservative Management	Rest, bracing, pain management with analgesics, physiotherapy	Mild to moderate fractures, no neurological deficits, stable fractures	Non-invasive, avoids surgical risks, allows natural healing
Vertebroplasty	Injection of bone cement into fractured vertebra to stabilize	Painful fractures not responding to conservative treatment, no neurological symptoms	Provides rapid pain relief, improves vertebral stability
Kyphoplasty	Balloon inserted to restore vertebral height, followed by cement injection	Fractures causing significant vertebral height loss, pain, or deformity	Restores vertebral height, reduces pain, improves mobility
Surgical Intervention	Spinal fusion, implants, decompression surgery for nerve involvement	Severe fractures, instability, neurological deficits, failure of other treatments	Corrects deformity, stabilizes spine, relieves nerve compression

**Table 6:** Summary of imaging modalities for diagnosing VCFs.

Modality	Characteristics	Benefits	Limitations
Radiograph	Standard imaging technique, often first-line for detecting fractures and alignment	- Quick and widely available - Low cost	- Limited in detecting subtle fractures - No soft tissue or bone marrow edema evaluation
CT including DECT	Detailed cross-sectional images of bone and vertebral structures	- Excellent bone detail - Quick acquisition - Detects complex fractures - DECT detects bone marrow edema and acute vs. chronic VCFs	- Higher radiation dose than radiographs - Limited soft tissue evaluation except DECT - DECT is less sensitive compared to MRI
MRI	Superior soft tissue contrast, gold standard for detecting bone marrow edema	- High sensitivity for detecting bone marrow edema and soft tissue injuries such as ligaments - No radiation	- Time-consuming - Expensive - Contraindicated in patients with certain pacemakers or metal implants
PET and Bone Scan	Detects metabolic activity and useful in identifying metastatic disease or infection, detects increased bone activity	- PET is highly sensitive for cancer and infection - Can differentiate benign vs. malignant fractures - Bone scans are sensitive for occult fractures	- High cost - Involves radiation exposure - Limited for purely traumatic fractures

## Data Availability

Not applicable.
